# Rapid communication of efforts to resolve differences or update variant interpretations in ClinVar through case-level data sharing

**DOI:** 10.1101/mcs.a003467

**Published:** 2018-10

**Authors:** Heidi Rehm

**Affiliations:** Center for Genomic Medicine, Massachusetts General Hospital, Boston, Massachusetts 02114, USA; The Broad Institute of MIT and Harvard, Cambridge, Massachusetts 02142, USA

In this issue of *Cold Spring Harbor Molecular Case Studies*, we have included the first publication (Grant et al. 2018) of a new article type, the “Variant Discrepancy Resolution.” This format has been created in response to the growth and utility of the ClinVar database as a repository of variant-level data (Landrum et al. 2018). The largest number of ClinVar submissions, >85%, are now coming from clinical laboratories that have voluntarily shared their interpretations of variants, as mechanisms to both crowdsource the enormous challenge of variant interpretation and ensure the most accurate interpretations are returned to patients. Behind each submission is often a rich body of evidence, some of which is published, but often valuable unpublished case data cannot be easily shared because of the lack of patient consent in the clinical testing process. Instead, laboratories are alerted to the potential for a different evidence base when their submitted variant interpretations show up as conflicting in ClinVar. These alerts prompt submitters to reach out and exchange this detailed case-level data, which can then be used to resolve the differences in interpretation or, in some cases, move a variant that is consistently classified as a variant of uncertain significance (VUS) up or down toward a pathogenic or benign interpretation.

In this issue, two clinical laboratories had both interpreted a variant as a VUS, but, upon contact by a patient whose fetus harbored the variant, they exchanged detailed case-level evidence that enabled the variant to move to a Likely Benign interpretation. This rapid exchange also led the family to continue the pregnancy without concern for an adverse outcome. Recent publications from ClinGen have also demonstrated the benefit of sharing unpublished data from clinical laboratories to resolve discrepancies or reclassify VUSs (Gelb et al. 2018; Kelly et al. 2018). The Editors of *Cold Spring Harbor Molecular Case Studies* encourage these interlaboratory exchanges and have generated an article type to allow this work to be recognized and more rapidly captured in a documented form that the community can access.

Clinical laboratories can obtain reports of their variant interpretation differences from the ClinVar Miner website (Henrie et al. 2018; https://clinvarminer.genetics.utah.edu/) or request a custom report from ClinGen (clingen@clinicalgenome.org), which will also highlight variants in which the submitter has an outlier interpretation compared to other submitters (Harrison et al. 2018). Studies have shown that most of the differences in interpretation can be resolved, with a large percentage due to the exchange of unpublished data (Garber et al. 2016; Harrison et al. 2017). We encourage the community to engage in interlaboratory exchange of case-level data and submit your work to *Cold Spring Harbor Molecular Case Studies*.

## Competing Interest Statement

The author has declared no competing interest.
